# Transpulmonary thermodilution for hemodynamic measurements in severely burned children

**DOI:** 10.1186/cc10147

**Published:** 2011-04-21

**Authors:** Ludwik K Branski, David N Herndon, Jaron F Byrd, Michael P Kinsky, Jong O Lee, Shawn P Fagan, Marc G Jeschke

**Affiliations:** 1 Shriners Hospitals for Children, 815 Market Street, Galveston, TX 77550, USA; 2 Department of Surgery, University of Texas Medical Branch, 301 University Boulevard, Galveston, TX 77555, USA; 3 Department of Anesthesiology, University of Texas Medical Branch, 301 University Boulevard, Galveston, TX 77555, USA; 4 Department of Surgery, Massachusetts General Hospital, Shriners Hospitals for Children, and Harvard Medical School, 25 Shattuck Street, Boston, MA 02115, USA; 5 Ross Tilley Burn Centre, Sunnybrook Health Sciences Centre, and Department of Surgery, Division of Plastic Surgery, University of Toronto, 2075 Bayview Avenue, Toronto, ON M4N 3M5, Canada

## Abstract

**Introduction:**

Monitoring of hemodynamic and volumetric parameters after severe burns is of critical importance. Pulmonary artery catheters, however, have been associated with many risks. Our aim was to show the feasibility of continuous monitoring with minimally invasive transpulmonary thermodilution (TPTD) in severely burned pediatric patients.

**Methods:**

This prospective cohort study was conducted in patients with severe burns over 40% of the total body surface area (TBSA) who were admitted to the hospital within 96 hours after sustaining the injury. TPTD measurements were performed using the PiCCO system (Pulsion Medical Systems, Munich, Germany). Cardiac Index (CI), Intrathoracic Blood Volume Index (ITBVI) (Stewart-Hamilton equation), Extravascular Lung Water Index (EVLWI) and Systemic Vascular Resistance Index (SVRI) measurements were recorded twice daily. Statistical analysis was performed using one-way repeated measures analysis of variance with the *post hoc *Bonferroni test for intra- and intergroup comparisons.

**Results:**

Seventy-nine patients with a mean age (±SD) of 9 ± 5 years and a mean TBSA burn (±SD) of 64% ± 20% were studied. CI significantly increased compared to level at admission and was highest 3 weeks postburn. ITBVI increased significantly starting at 8 days postburn. SVRI continuously decreased early in the perioperative burn period. EVLWI increased significantly starting at 9 days postburn. Young children (0 to 5 years old) had a significantly increased EVLWI and decreased ITBVI compared to older children (12 to 18 years old). EVLWI was significantly higher in patients who did not survive burn injury.

**Conclusions:**

Continuous PiCCO measurements were performed for the first time in a large cohort of severely burned pediatric patients. The results suggest that hyperdynamic circulation begins within the first week after burn injury and continues throughout the entire intensive care unit stay.

## Introduction

Large burns over greater than one-third of the total body surface area (TBSA) result in a massive inflammatory response, which in turn causes severe and unique hemodynamic and cardiovascular challenges. Early excision of necrotic tissue and prompt coverage temper the postburn hypermetabolic response, decrease excess fluid loss and ultimately lead to improved survival [1-3]. Still, continued hemodynamic support with appropriate fluid resuscitation and administration of cardiovascular agents are needed in the early postburn period to oppose hypervolemia, alterations in afterload and myocardial depression [4-7], which can accelerate organ dysfunction [8].

Invasive hemodynamic monitoring via a pulmonary artery catheter (PAC) permits the direct and continuous measurement of central venous pressure (CVP), pulmonary capillary occlusion pressure, cardiac output (CO), Systemic Vascular Resistance Index (SVRI) and oxygen delivery and consumption. However, the PAC is highly invasive and associated with substantial risks that often outweigh its benefits [9]. To overcome the disadvantages of the PAC, less invasive techniques have been developed. The PiCCO catheter (Pulsion Medical Systems, Munich, Germany) combines advanced hemodynamic monitoring and volumetric measures without the necessity of a right heart catheterization. It utilizes transpulmonary thermodilution (TPTD), in which a cold saline bolus is injected into the central venous circulation, and the subsequent change in blood temperature is measured by a thermistor-tipped arterial catheter, allowing for the determination of CO [10-12]. Additionally, TPTD estimates global end-diastolic volume and Intrathoracic Blood Volume Index (ITBVI), indicators of cardiac preload, and Extravascular Lung Water Index (EVLWI), an index of pulmonary edema [13]. The use of TPTD goal-directed therapy based on ITBVI and EVLWI measurements in critically ill patients has been studied in various prospective trials and has shown promising results [14]. Only one prospective randomized study that compared goal-directed therapy guided by TPTD measurements with standard care (Baxter formula) in burn shock management has been performed in adult burn patients [11].

At the Shriners Hospitals for Children in Galveston, TX, USA, TPTD has been the standard of care for hemodynamic monitoring of children with severe burns over 40% of the TBSA. The goals of this study were to report the hemodynamic and volumetric status in severely burned children within the first 3 weeks postburn, to identify differences in hemodynamic parameters between different age groups and to identify differences in hemodynamic parameters between survivors and nonsurvivors.

## Materials and methods

Severely burned children admitted to the Shriners Hospitals for Children between December 2005 and March 2008 were considered for entry into this study. Permission for conducting the study was obtained from the Institutional Review Board at the University of Texas Medical Branch, Galveston, TX, USA (protocol 08-289). Informed written consent was obtained in all cases. The following inclusion criteria were used: burn size equal to or exceeding 40% of TBSA and at least 30% TBSA full thickness burn, patients admitted within 120 hours of injury and patients not septic at admission. Exclusion criteria included any kind of cardiopulmonary illness.

All patients were weighed on admission, and calculation of all indexed values was based on the initial burn size and the body surface area of the individual patient. Analgesia and sedation were performed according to routine guidelines followed at our institutions. If mechanical ventilation was required, initial ventilator settings included a pressure-controlled mode of ventilation, a frequency of 10 to 15 breaths/minute, inspiration/expiration time of 1:2 and initial positive end-expiratory pressure (PEEP) of 4 cmH_2_O. PEEP was adjusted according to the pulmonary function and oxygenation level of the patient. All patients underwent staged early excision and grafting with autografts, allografts or both between 48 and 72 hours postburn and at approximately weekly intervals thereafter. Expanded autograft (meshed 1:4) with allograft overlay was applied to as much of the burn area as was possible to cover. The rest of the wound area was covered with unexpanded fresh allograft (meshed 1:1.5). Donor sites were recropped when healed, and the allograft was surgically excised and consecutively replaced with autograft skin.

### Demographics

Mortality rates, length of intensive care unit (ICU) stay, cumulative length of hospital stay based on 95% healing of grafts, total number of procedures performed during acute admission and total operating room (OR) time were recorded. Weights were measured within 5 days of admission and at discharge using standard clinical scales. The clinical scales were calibrated monthly.

### PiCCO measurements

All patients had central venous (inferior or superior vena cava) and arterial (brachial, radial or femoral artery) access placed upon initial admission. TPTD measurements were performed using the Pulsiocath 3- or 4-French thermistor-tipped catheter (Pulsion Medical Systems, Munich, Germany). Cardiac Index (CI), ITBVI and ELWI were determined using an injection of 10 mL of cooled saline solution (0°C to 6°C) into the central venous catheter. SVRI was calculated based on measured CO, mean arterial pressure (MAP) and CVP. Injections were performed manually and were not coordinated with the respiratory cycle. Measurements were taken at least twice daily. Each procedure consisted of three injections via the venous access, and all saline boluses were administered within a maximum time span of 10 minutes. Results were calculated as the mean of these three consecutive measurements. Heart rate (HR), MAP and CVP were calculated on the basis of the aforementioned variables or recorded directly by the hardware at the same time points as the thermal bolus injections. Data were recorded and exported to a personal computer with PICCO-VoLEF-WIN software (version 4.0; Pulsion Medical Systems) combined with the Pulsion PICCOPlus device (PC 8100 software version V6.0; Pulsion Medical Systems).

### Statistics

For interindividual comparisons, all flow-related or volume-related variables were normalized to TBSA. Continuous values were compared using Student's *t*-test or the Mann-Whitney *U *test, depending on their distributional properties. To test the influence of time on the hemodynamic and volumetric variables, a two-way analysis of variance was performed to determine the statistical significance of the change over time of each of the variables and the influence of treatment. When a difference was detected, *post hoc *analysis was performed using the Bonferroni correction. Differences in proportions, such as mortality rate, infection rate and incidence of sepsis were compared using the χ^2 ^test. In all cases, *P *< 0.05 was considered statistically significant.

## Results

Between December 2005 and September 2008, 79 acutely burned children were enrolled into the study. The demographics of the patient groups are listed in Table 1 (for the complete cohort), Table 2 (for different age groups) and Table 3 (for survivors and nonsurvivors).

**Table 1:** Entire cohort demographics^a^

Demographic variable	Statistics
Number of patients	79
Age, yr	9.2 (9.3)
TBSA burn, %	64.0 (35.0)
TBSA full thickness burn, %	50.0 (45.5)
Type of burn, %	
Flame	70%
Scald	25%
Other	5%
Male:female ratio	2.3:1
Length of stay, days	28.6 (23.6)
Survivors, %	80%

^a^TBSA, total body surface area. Data are presented as medians (interquartile range).

**Table 2:** Age group demographics^a^

Age group	0 to 4.9 years	5 to 11.9 years	12 to 18 years	*P *value
Number of patients	21	31	27	ns
Age, years	3 (2)	8 (4)	15 (3)	< 0.05
Time from burn to admission, hours	44 (36)	29 (45)	42 (67)	ns
TBSA burn, %	64 (41)	61 (31)	73 (32)	ns
TBSA third-degree burn, %	60 (43)	48 (33)	53 (51)	ns
Male:female ratio	2:1	5.3:1	1.3:1	ns
Length of stay (days)	29 (25)	28 (22)	31 (23)	ns

^a^TBSA, total body surface area; ns, not significant. Data are presented as medians (interquartile range).

**Table 3:** Demographics of survivors versus nonsurvivors^a^

Group	Survivors	Nonsurvivors	*P *value
Number of patients	64	15	
Age, yr	8 (9)	12 (8)	ns
Time from burn to admission, hours	33 (31)	61 (71)	ns
TBSA burn, %	58 (30)	87 (11)	< 0.001
TBSA third-degree burn, %	46 (41)	81 (16)	< 0.001
Male:female ratio	1.7:1	1.5:1	ns
Length of stay, days	28 (19)	33 (39)	ns

^a^TBSA, total body surface area; ns, not significant. Data are presented as medians (interquartile range).

### Complete cohort

**Figure 1:**
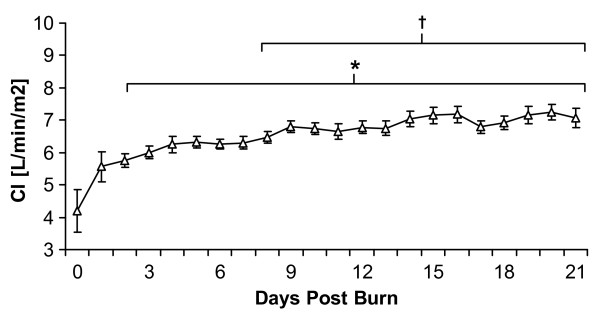
**Cardiac Index (CI) levels for the entire patient cohort between burn (day 0) and day 21 postburn**. Data are expressed as means ± standard error of the mean. *P < 0.05 versus day 0. †*P *< 0.05 versus day 1.

**Figure 2:**
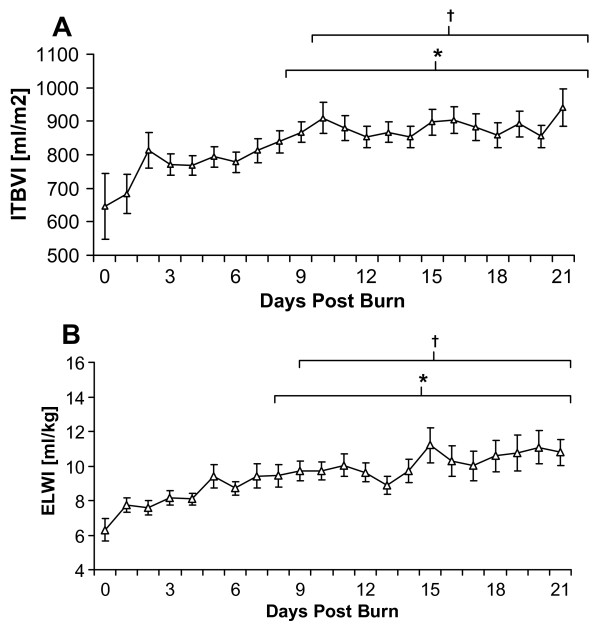
**Intrathoracic blood volume and extravascular lung water**. **(A)**Intrathoracic Blood Volume Index (ITBVI) levels and **(B) **Extravascular Lung Water Index (ELWI) levels for the entire patient cohort between burn (day 0) and day 21 postburn. Data are expressed as means ± standard error of the mean. *P < 0.05 versus day 0. †*P *< 0.05 versus day 1.

**Figure 3:**
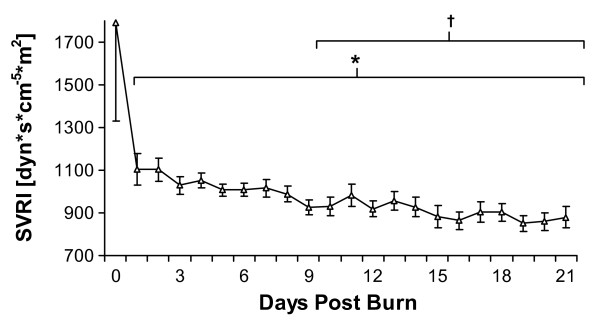
**Systemic Vascular Resistance Index (SVRI) levels for the entire patient cohort between burn (day 0) and day 21 postburn**. Data are expressed as means ± standard error of the mean. *P < 0.05 versus day 0. †*P *< 0.05 versus day 1.

In the complete cohort of severely burned children, MAP remained relatively unchanged with a mean value of 85 mmHg, CVP increased after the initial loading phase and then gradually declined during the remainder of the measurement period and HR remained elevated with tachycardia during the entire acute ICU stay (data not shown). CI was significantly increased compared to admission values after the second day of admission and, overall, continuously increased during the entire measurement period (Figure 1). ITBVI and EVLWI measurements showed similar patterns: a gradual increase over the entire measurement period, reaching significance when compared to day 0 or day 1 at 8 or 9 days postburn, respectively (Figure 2A and 2B). SVRI values demonstrated a continuous decrease during the measurement period, also reaching significance after 9 days postburn when compared to the first day postburn (Figure 3).

### Age groups

**Figure 4:**
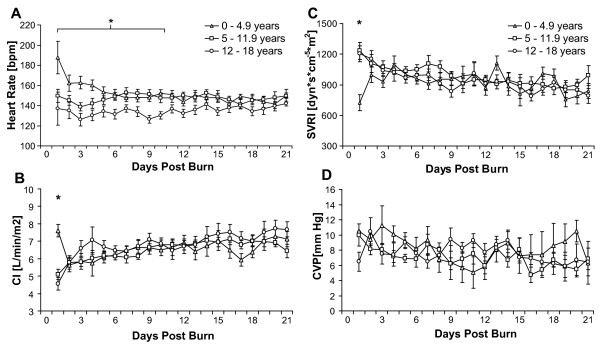
**Heart rate, cardiac index, vascular resistance and venous pressure by age group**. **(A) **Heart rate, **(B) **Cardiac Index, **(C) **Systemic Vascular Resistance Index and **(D)**central venous pressure (CVP) measurements in three different age groups between burn (day 0) and day 21 postburn. Data are expressed as means ± standard error of the mean. *P < 0.05 for youngest (0 to 4.9 years) versus oldest (12 to 18 years) age groups.

**Figure 5:**
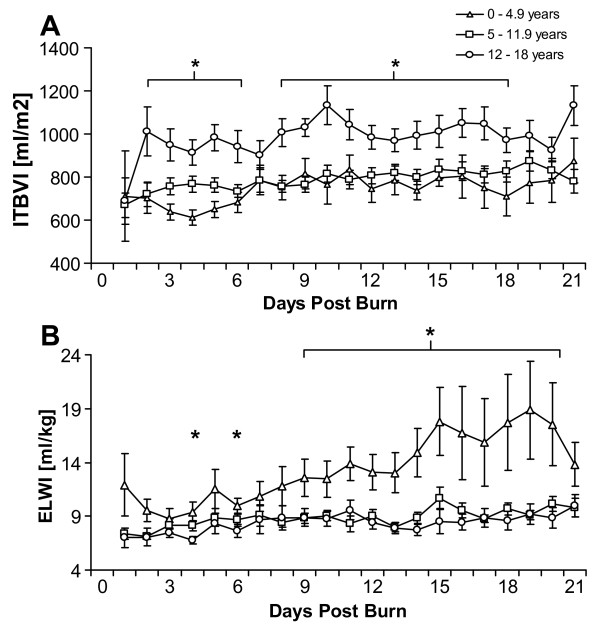
**Intrathoracic blood volume and extravascular lung water by age group**. **(A) **Intrathoracic Blood Volume Index and **(B) **Extravascular Lung Water Index measurements in three different age groups between burn (day 0) and day 21 postburn. Data are expressed as means ± standard error of the mean. *P < 0.05 for youngest (0 to 4.9 years) versus oldest (12 to 18 years) age groups.

Patients were divided into three age groups (Table 2). HR was significantly increased in the youngest children compared to the oldest age group until 10 days postburn (Figure 4A). No significant differences in CI measurements were observed between age groups after day 1 postburn (Figure 4B). SVRI initially was significantly lower in the youngest age group compared to both older patient groups. This difference, however, was not sustained after the end of the volume loading phase on day 2 postburn (Figure 4C). CVP in the youngest patient group compared to older children showed increased values throughout the measurement period. The differences, however, reached significance levels only sporadically (Figure 4D). ITBVI and EVLWI showed an opposing pattern in the youngest children versus the oldest patient group: a significant increase in ITBVI was observed in the oldest patient group compared to the youngest group (Figure 5A), while EVLWI displayed significantly higher values in the youngest patient group compared with the older patient group throughout most of the measurement period (Figure 5B).

### Survivors versus nonsurvivors

**Figure 6:**
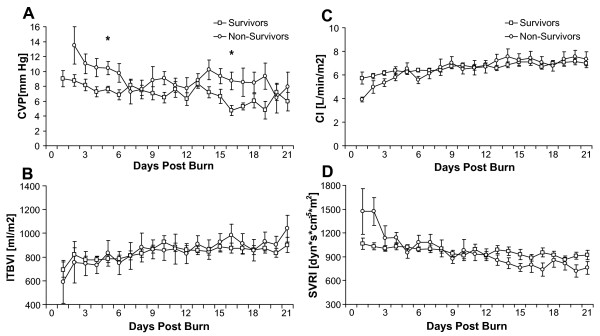
**Hemodynamic and volumetric parameters in survivors versus nonsurvivors**. **(A) **Central venous pressure, **(B) **ITBVI, **(C) **CI and **(D) **SVRI measurements in survivors versus nonsurvivors between burn (day 0) and day 21 postburn. Data are expressed as means ± standard error of the mean. *P < 0.05 for survivors versus nonsurvivors.

**Figure 7:**
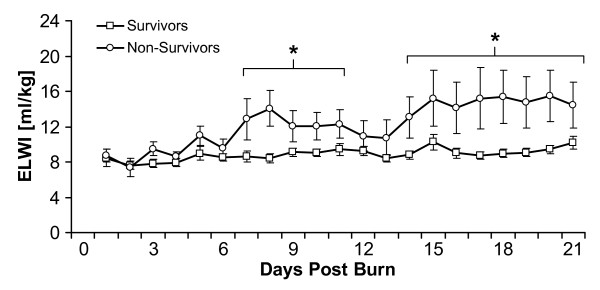
**Extravascular Lung Water Index measurements in survivors versus nonsurvivors between burn (day 0) and day 21 postburn**. Data are expressed as means ± standard error of the mean. *P < 0.05 for survivors versus nonsurvivors.

Patients were subdivided into those who survived and those who died during the acute stay (Table 3). MAP and preload and afterload parameters showed no significant differences between groups (Figure 6A to 6D). On the other hand, EVLWI was significantly higher in the nonsurvivors compared to the survivors (Figure 7).

### Complications

One child developed an arterial embolism in the left leg approximately 1 week after arterial catheter placement. However, since the patient also had coagulopathy, it is not clear whether the catheter placement or the coagulopathy caused the embolism.

## Discussion

Early excision and debridement of burn-injured tissues, coupled with prompt coverage, are an integral part of burn management [1-3]. Adequate fluid resuscitation in the first 24 to 48 hours postburn to overcome hypovolemia and restore hemodynamic and cardiovascular function remains a pivotal part of acute burn care [15]. Formulas for the calculation of resuscitation fluid requirements (Parkland, Brooke and Galveston formulas) have been established, and the needs of the individual patient are addressed based on constant reassessment of urinary output and volume status [8]. For the first time in a large cohort of severely burned children, hemodynamic and volumetric parameters were assessed for the first 3 weeks of ICU stay. Patterns of hemodynamic measurements were established using the PiCCO catheter, a novel technology based on TPTD.

In the phase of early resuscitation after a severe burn, it is of paramount importance to promptly restore vascular volume and to preserve tissue perfusion but minimize tissue edema [16]. The primary goal of therapy is to replace the massive intravascular volume loss due to the pathophysiological response to thermal injury. Resuscitation formulas such as Evans, Brooke and Parkland have been developed over the past decades as initial guides for volume replacement therapy applied to preserve adequate organ perfusion [15]. After the first 72 hours postburn, fluid management needs to be frequently reevaluated to avoid hypovolemia, hypervolemia and edema or organ dysfunction. Clinical monitoring of burn shock resuscitation and general fluid management has traditionally been carried out on the basis of the clinical assessment of cardiovascular status, urine output and biochemical parameters as indicators of vital organ perfusion. HR, blood pressure, CVP, electrocardiographic recording and baseline laboratory measurements (complete blood count, electrolytes, glucose, albumin and base deficit [17]) are the primary modalities for monitoring the volumetric and cardiovascular status in any patient. Fluid balance during burn shock resuscitation is typically monitored by measuring hourly urine output via an indwelling bladder catheter. A general recommendation during the early postburn period is to administer volume support to produce urinary output between 30 and 50 mL/hour in adults [18] and between 1.0 and 1.5 mL/kg/hour in patients weighing less than 30 kg [19]. It has been demonstrated, however, that overresuscitation is associated with adverse outcome and increased mortality in burn patients [19].

Invasive hemodynamic monitoring has been used in ICU settings for the past three decades. The advent of pulmonary artery catheterization permitted the direct measurement of CVP, pulmonary capillary wedge pressure, CO, SVRI, oxygen delivery and oxygen consumption. PAC-guided therapy has been studied most extensively in trauma and critically ill adult surgical patients. Although controversial, some suggest that hemodynamic data derived from the PAC are beneficial to ascertain cardiovascular performance in certain situations, such as in patients with inadequate noninvasive monitoring or when end points of resuscitation cannot be clearly defined [20]. Investigators in two studies reported that PAC-guided monitoring with resuscitation to hyperdynamic end points decreased ICU stay, ventilator days and incidence of organ failure when compared to patients resuscitated to normal hemodynamic values [21,22]. In burn patients, studies of the use of PAC for goal-directed burn shock resuscitation have shown a benefit of more aggressive resuscitation to hyperdynamic end points, with decreased mortality and ICU stay [23]. However, the general practicability, risk-benefit ratio and lack of mortality reduction associated with using PAC have been widely criticized. In the past decade, its use in the United States has decreased significantly [9]. So far, no prospective study of the use of goal-directed PAC therapy has been conducted in a pediatric burn population.

The PiCCO catheter was developed in Germany by Ulrich Pfeiffer in the 1980s [24]. Briefly, it represents a combination of two techniques for advanced hemodynamic and volumetric management without the necessity of a right heart catheterization. It utilizes TPTD, in which a cold saline bolus is injected into the central venous circulation, and the subsequent change in blood temperature is picked up by a thermistor-tipped arterial catheter [25]. CO is calculated by means of the Stewart-Hamilton equation using data derived from the area of the TPTD curve. Stroke volume variation and SVRI data are derived from the arterial pulse contour. ITBVI and EVLWI measurements are derived from, respectively, (1) the mean transit time and CO and (2) the down slope time of the thermodilution curve. The limitations of this technology include the presence of an intracardiac right-left shunt [25]. In our patient cohort, there was no evidence of intracardiac shunts (data not shown).

There is limited information on goal-directed therapy using TPTD measurements in burn patients. Holm *et al. *[11] used TPTD goal-directed therapy for the initial resuscitation of burn shock in adult burn patients compared to controls who were resuscitated according to the Baxter formula. TPTD-directed resuscitation was associated with increased fluid requirements compared to controls during the first 48 hours following burn injury. One conclusion might be that TPTD results in more aggressive fluid infusion, which could be detrimental. However, TPTD was shown to reduce the incidence of hypovolemia compared to the Baxter formula, and EVLWI was not different [11]. So far, no randomized clinical trials have been performed using TPTD-guided therapy for acute burn shock in severely burned pediatric patients. Furthermore, there have been no reports on the continuous use of TPTD for hemodynamic and cardiovascular monitoring in burn patients during their entire ICU stay.

In the present study, the PiCCO catheter was used to measure critical hemodynamic and volumetric parameters over time following severe burn injury in pediatric patients. We sought to determine the influence of age on the hemodynamic response to burn injury, as well as how information obtained by the PiCCO catheter could be used as a predictive tool for determining mortality.

With regard to the entire patient cohort, CO continuously increased over the entire study period. This hypermetabolic circulation has been shown to persist for more than 2 years postburn [26]. The product of increased HR and decreased SVRI results in the flow phase postburn, which has been demonstrated to have a major impact on burn patient outcomes.

We were able to demonstrate significant differences between our youngest patients (mean age, 3 years) and the oldest group (mean age, 15 years). The youngest patient group showed markedly elevated EVLWI (up to 25 mL/kg in some cases) compared to the older patients. Our results are in agreement with those of Schiffmann *et al. *[27], who demonstrated that critically ill infants had mean EVLWI of over 27 mL/kg. These authors speculated that increased EVLWI was related to the severity of the underlying disease. However, they also acknowledged that since normal EVLWI values are not defined for infants, apart from single case reports [28], the underlying cause remains unclear.

The effect of an increase in EVLWI on mortality is well-established in ICU patients [29]. Furthermore, protocols using EVLWI as a monitor to guide volume and other cardiovascular support have been shown to decrease length of ICU stay [30] and mortality when employing a fluid restriction approach [29]. In general, fluid restriction therapy in ICU patients with acute lung injury improves lung function and shortens the duration of mechanical ventilation [31]. The key finding in our large cohort of severely burned children is consistent with that of Eisenberg *et al. *[29], who showed that increased EVLWI is associated with mortality. It remains to be determined whether goal-directed approaches using EVLWI as a primary end point to direct fluid support in severely burn-injured children will indeed have an influence on mortality. The use of a normalized and validated morbidity score, such as the Pediatric Logistic Organ Dysfunction score [32,33], to support an interpretation of organ failure is another way to determine how the use of the PiCCO catheter can be used as a predictor of morbidity and mortality. A prospective study is currently underway at our institution to determine the effects of the use of TPTD on clinical outcomes, including organ function.

## Conclusions

Burn patients show an impairment of ventricular compliance consistent with experimental models of burn injury [34-38], and this impairment is more pronounced in the youngest patient group. After the initial volume loading, ongoing fluid replacement schemes may not be adequate for the very young patient (under age 3 years), as seen in this study with regard to the measurement of EVLWI. Overall, TPTD measurement is a rapid, safe and easy-to-install method for minimally invasive hemodynamic monitoring. The obtained CO and preload and afterload variables have been validated in multiple studies [12,39-42]. Compared to the PAC, the PiCCO methodology may represent a superior method to direct fluid therapy support, since ITBVI is a more sensitive and specific indicator of cardiac preload than pulmonary artery occlusion pressure or CVP [42]. This is likely due to the higher preload specificity of volume versus pressure.

## Key messages

• Key volumetric and hemodynamic parameters such as CO, ITBVI, EVLWI and SVRI can be measured in severely burned pediatric patients with the TPTD technique, which is less invasive than PAC techniques.

• Severely burned children up to 5 years old have significantly increased EVLWI levels and significantly decreased ITBVI values compared to those of children between 12 and 18 years of age, which underscores the importance of tightly controlled fluid management in the burn injured child.

• The hyperdynamic state in a burned patient begins within the first week after burn injury and continues throughout the entire ICU stay.

## Abbreviations

CI: cardiac index; CVP: central venous pressure; EVLWI: Extravascular Lung Water Index; ICU: intensive care unit; ITBVI: Intrathoracic Blood Volume Index; MAP: mean arterial pressure; OR: operating room; PAC: pulmonary artery catheter; PEEP: positive end-expiratory pressure; SVRI: Systemic Vascular Resistance Index; TBSA: total body surface area; TPTD: transpulmonary thermodilution.

## Competing interests

The authors declare that they have no competing interests.

## Authors' contributions

LKB participated in the design of the study, collected the data and drafted the manuscript. DNH conceived of the study and participated in its design and coordination as well as manuscript preparation. JFB collected data and participated in manuscript preparation. MK participated in manuscript preparation and data analysis. JOL, SPF and MGJ participated in data collection, study design and manuscript preparation. DNH and MGJ did study coordination. All authors read and approved the final manuscript.
